# Behavioral and Subjective Music Perception and Appreciation in Adults with Hearing Aids, Cochlear Implants, and Typical Hearing

**DOI:** 10.3390/audiolres16040113

**Published:** 2026-08-04

**Authors:** Stephanie Fowler-Brookman, Andrea D. Warner-Czyz

**Affiliations:** 1 School of Behavioral and Brain Sciences, The University of Texas at Dallas, Richardson, TX 75080, USA; warnerczyz@utdallas.edu; 2 Callier Center for Communication Disorders, Dallas, TX 75235, USA

**Keywords:** behavioral music perception, subjective music abilities, subjective music importance, adults, deaf or hard of hearing, hearing aids, cochlear implants, typical hearing

## Abstract

**Background/Objectives**: Even with the use of hearing aid or cochlear implant technologies, adults who are deaf or hard of hearing (DHH) exhibit difficulty perceiving musical characteristics compared to peers with typical hearing, but do not necessarily report a consistent decrease in appreciation of musical activities. This study addresses a gap in the literature by examining the effect of both hearing status (i.e., DHH versus typical hearing) and hearing technology (i.e., hearing aids versus cochlear implants) on both behavioral and subjective music abilities and ratings of music importance. **Methods**: Adults with typical hearing (n = 20), hearing aids (n = 10), or cochlear implants (n = 10) completed tasks on (a) behavioral measures of music perception including pitch perception, melody recognition, and timbre recognition; and (b) the Music-related Quality of Life questionnaire to assess subjective ratings of music abilities and music importance. **Results**: Adults with typical hearing outperformed the cochlear implant group on all behavioral music perception tasks, but they performed similarly to the hearing aid group. Although the two DHH groups subjectively rated their musical abilities less positively than adults with typical hearing, all three groups appraised the importance of music in their lives similarly. **Conclusions**: Music importance and appreciation do not depend on music abilities, implying that clinicians and researchers should incorporate metrics of subjective music quality of life to comprehensively assess and treat adults who are DHH to foster more positive quality of life.

## 1. Introduction

Music—the second most important sound besides speech—plays an important cultural, social, and emotional role in the lives of individuals across their lifespan [1,2]. Music can be used to heighten both negative and positive emotions [3,4]; to create social bonds and establish independent identities [5,6]; and to elevate religious services and lifecycle ceremonies [7]. In fact, Gasser [8] identifies seven distinct uses for music universal to most cultures: overall cultural identity, social aspects, religious services, and as part of work, defense, education, and entertainment. Because music covers a large swath of functions and cultures, its accessibility to individuals who are deaf or hard of hearing (DHH) may be critical to fostering positive quality of life and active engagement within their broader societal and cultural contexts [9,10,11].

In Western societies, music composition relies heavily on a precise relationship among different pitches, which can sound consonant or discordant [12,13]. In addition, differences in intensity generate a sense of drama or enhance the main message of a song. Finally, music is often composed with multiple instruments layered together, which generally increases the complexity of the music for the listener. Timbre perception, or quality of the sound, supports our capacity to parse these layered sounds and instruments. Popular music typically also includes lyrics. The relationship among pitches, intensity, timbre, and lyrics in a song is complex, but individuals who are DHH may experience difficulty perceiving or appreciating these intentional decisions by the composer.

Individuals who are DHH may have trouble perceiving some frequency regions more than others (e.g., sloping hearing loss in which hearing sensitivity is better for low-frequency than high-frequency sounds) [14,15]. At a biological level, this disrupts relationships between pitches, such that a listener may perceive two pitches as discordant when the composer intended for them to be concordant.

Hearing technology may also influence music perception beyond anatomical and physiological changes in the auditory system. Individuals who are DHH often begin their hearing health journey with the use of hearing aids (HAs). HAs provide a robust solution for treating a range of hearing levels, types, and configurations [16]. However, to prioritize speech processing, HAs include digital features such as compression in the intensity and frequency domains; noise reduction algorithms; directionality; and increased automatic processing to change settings based on their auditory environment. These strategies benefit speech recognition in noise but can disrupt the relationship of the pitch and intensity of the music to which an individual may be listening, potentially leading to poorer audibility, increased distortion, and greater dissonance [17].

For some individuals, a HA may not provide adequate auditory access and resolution of speech, music, and other aspects of the auditory environment. That is, music perception demands high spectral resolution across a broader frequency range than speech and HAs are more likely to saturate at higher frequencies. Thus, other hearing technologies such as a cochlear implant (CI) are a more appropriate option. Although a CI can yield improved speech recognition outcomes for individuals with moderate to profound hearing levels [18,19], the CI has inherent limitations in pitch and auditory information processing that negatively affect music perception. For example, a CI electrode array has reduced spectral fidelity with its 12 to 24 electrode contacts, which is reduces the representation of 22,000 hair cells and 3500 inner hair cells [14]. In addition, the place-pitch mismatch resulting from the misalignment of the location of each electrode and its frequency band relative to the physical area of the cochlea likely is not optimally tuned to that frequency [20,21]. Finally, a CI provides poorer representation of the temporal envelope due to lack of low-pass filtering of the temporal envelope above 150–220 Hz, which also negatively affects the perception of pitch and melody [22]. Both reduced spectral fidelity and place-pitch mismatch could lead to significant perceptual differences in discrete pitch understanding.

Hearing loss and hearing technology limitations may negatively affect music perception. Behavioral measures of music perception tests often focus on discrete characteristics of music perception, generally categorized as spectral, temporal, and spectral–temporal aspects of music. Spectral musical elements typically include pitch (e.g., high- or low-frequency sounds) and melody (i.e., a sequence of pitches). Temporal aspects of music often include rhythm (i.e., temporal patterns of sounds) and tempo (i.e., speed of a musical piece). Spectral–temporal components of music refer to timbre (i.e., discriminating instruments). The following section summarizes research exploring the effect of hearing loss on these behavioral measures of music perception.

### 1.1. Effect of Hearing Loss on Behavioral Measures of Music Perception

Adults who are DHH using HAs vary in performance on music perception tasks and appreciation of music with hearing technology [9]. Variability in performance may reflect the heterogeneity of deafness relative to participant characteristics (e.g., chronologic age, sample size, musicianship, control group), audiologic history (e.g., level and configuration of hearing thresholds, hair cell survival, etiology, onset of hearing loss), hearing technology (e.g., HA, CI, device configuration), and test construction (e.g., pitch perception assessed as accuracy perceiving quarter-, half-, and full-octave differences vs. number of semitones needed to discriminate sounds).

Few studies investigate behavioral music perception in adults wearing HAs [23,24]. For spectrally based musical elements, Looi et al. [23] found that HA users achieve high levels of pitch perception for half- and full-octave differences (84–90% correct), with lower accuracy for quarter-octave differences (75%) [23] and excellent melody recognition (91%). However, Uys and van Dijk [24] reported less positive outcomes, with HA users scoring 74% correct on a pitch identification task, 68% correct on a pitch discrimination task, and 68% correct on a melody identification task compared to scores of 80–93% in listeners with typical hearing (TH). HA users exhibit good to excellent rhythm discrimination, depending on the task parameters (75–94% correct), but scores lag behind those of adults with TH (98% correct) [24,25]. Assessment of timbre perception generally supports better scores with single-instrument (59–69%) versus multiple-instrument (52–54%) stimuli [24,25].

CI user performance also varies by characteristics of music perception. Adults with CIs perform similarly to adults with HAs [23] and adults with TH in the temporal aspects of music [25,26,27,28,29,30,31]. For example, Looi et al. found that both HA and CI users scored >90% correct on a same/different rhythm identification task [23] and Brockmeier and colleagues reported that adult CI listeners score, on average, 79% correct and adults with TH score 84% correct on a same/different rhythm discrimination task [31]. Both CI and TH groups can identify tempo discrimination with four to six beats per minute [30]. However, CI users exhibit poorer performance when tested on spectral characteristics of music, which includes pitch discrimination and melody identification [32,33,34,35,36]. For example, Gfeller et al. [36] found that, compared to TH listeners, CI users need larger intervals to discriminate pitch (i.e., *M* = 7.6 semitones and 1.1 semitones in the CI and TH groups, respectively), recognize fewer melodies (i.e., 12.6% versus 55.1% in the CI and TH groups respectively), and have greater intragroup variability in performance. More recent studies estimate that the difference limen for CI users approximates 3 semitones [35,37].

Few studies directly compare semitone-based pitch discrimination in both DHH groups. Looi et al. found the HA group to achieve significantly better accuracy (~20% better) for quarter-octaves, half-octaves, and full octaves than the CI group [23]. Additionally, listeners with CIs and TH both identify changes in pitch, but differ in ranking of pitch distortion levels in a melody [38], suggesting differences in place-pitch intervals in CI processing relative to TH. Finally, adult CI users and HA users perform similarly to each other on timbre-based tasks [23], but underperform relative to TH listeners, with mean performances on an instrument identification task consistently falling in the 40–50% range compared to 91–97% in adults with TH [15,23,34,35,37,39].

Research to date suggests that individuals who are DHH using hearing technology have relatively good preservation of temporal cues but poorer representation of spectral and spectral–temporal aspects of music. However, the influence of being DHH or using HAs or CIs on subjective ratings of music appreciation, engagement, and enjoyment has received less attention.

### 1.2. Effect of Hearing Loss on Music Appreciation and Engagement

Behavioral measures of music perception may not capture the effect of hearing loss and hearing technology on subjective aspects of music such as music appreciation and music-related quality of life. Subjective questionnaires typically include items focused on engagement with music (e.g., how many hours per week does the individual listen to music), self-reported music skills (e.g., can the individual perceive different pitches), and satisfaction with music overall (e.g., does the individual enjoy music). Various factors can affect music appreciation and engagement, including general interest in music and training with music.

Adults with HAs value the quality of music, with more than 80% rating it as very or extremely important to them [40]. Looi and colleagues [27] explored music listening and appreciation in adult HA users with mild, moderate, and severe levels of hearing loss, asking them to rate music engagement and enjoyment on a 100-point scale with higher values reflective of greater music engagement or enjoyment. Results show that the overall group continued to engage with music (*M* = 69.6), though the group with severe hearing levels indicated they spent less time listening to music (*M* = 51.2 vs. 74.8 and 71.3 for the severe, moderate, and mild groups, respectively). The groups, on average, reported that they enjoyed listening to music, but those with more severe hearing loss levels rated music enjoyment less positively (*M* = 68.4) than those with moderate (*M* = 81.3) or mild (*M* = 76.2) hearing loss levels. Similarly, studies indicate there are no significant differences in the frequency of music engagement, the importance of music, or enjoyment of music in adults with CIs vs. TH [41,42]. However, CI users vary considerably in their enjoyment of music, with 38 to 74% of adult CI users enjoying listening to music [43,44] and nearly half continuing to listen to music even if they are disappointed in how the music sounds after cochlear implantation [45].

### 1.3. Relationship Between Music Perception and Music Appreciation and Engagement

Although music perception abilities may contribute to music appreciation and satisfaction, the association between behavioral and subjective measures is influenced by personal experience and interest in music, often resulting in a dissociation between music perception abilities, engagement, and enjoyment. The disconnect between music perception and enjoyment persists in adults [10,37,41,42,45,46,47,48]. These studies in combination highlight the need to reject the assumption that music engagement and enjoyment in adult CI users depend on the presence of good music perception skills.

### 1.4. Purpose of This Study

To date, several studies examine music perception in adults who are DHH, but only a small number of studies compare across different hearing technologies [23,49,50,51]. This limits our understanding of the representation of music in acoustic versus electric signal processing in HAs and CIs. Moreover, the study by Looi et al. [23] asked participants to complete behavioral measures of music perception (e.g., pitch perception) without addressing subjective aspects of music perception such as self-perceived music abilities and the importance of music to listeners. Two studies incorporated behavioral and subjective measures of music perception, engagement, and enjoyment, but they compared listeners with TH and CI without including HA users in their comparisons [31,37]. Inclusion of both behavioral and subjective measures affords information for counseling relative to the reality of enjoying music with different types of hearing technologies relative to actual music perception abilities.

This study aims to address these gaps in the literature to investigate whether group differences on behavioral and subjective measures emerge based on the auditory status of the individual by exploring outcomes in three groups of adults—TH listeners, HA users, and CI users. By comparing these groups under one methodological approach, differences in performance and self-report can be elicited to better understand the musical experiences of individuals with hearing loss. Thus, this study asks the following three research questions:

1. What is the effect of hearing status on behavioral measures of music perception (i.e., pitch perception, instrument identification, familiar and unfamiliar melody recognition)? We anticipate that the TH group will outperform the HA group, who will in turn outscore the CI group due to the advantage of acoustic versus electric hearing relative to music perception. We do not expect task-based differences because all four tasks rely on spectral fidelity.
2. What is the effect of hearing status on self-reported music perception (i.e., music abilities) and music’s importance? Few studies examine self-reported music abilities, so this reflects an exploratory part of this research question. Based on previous studies, we hypothesize that the TH group will report slightly more music engagement than the HA group, which will report higher levels of engagement than the CI group. We expect all three groups will report similar levels of the importance of music.
3. What is the relationship between behavioral and subjective music perception in adults with TH, HA, or CI? We do not anticipate any significant correlations between behavioral and subjective music skills in the TH, HA, or CI groups.

## 2. Materials and Methods

### 2.1. Ethical Approval

The study was conducted in accordance with the Declaration of Helsinki and approved by the Institutional Review Board of The University of Texas at Dallas (IRB 18-146, initially approved 17 September 2018). Informed consent was obtained from all subjects involved in the study either in writing or online.

### 2.2. Participants

Adult participants were recruited from clinical sites and support groups in the Dallas-Fort Worth Metroplex and online support groups targeting musicians, individuals who are DHH, or both. Table 1 summarizes participant characteristics. Participants in the TH group (*n* = 20, 60% female) ranged in age from 22 to 37 years and had audiometric thresholds no poorer than 25 dB HL between 500 and 4000 Hz bilaterally. The HA group (*n* = 10, 80% female), whose ages spanned from 20 to 39 years, all had post-lingual hearing loss (i.e., onset after 3 years of age). All participants in the HA group wore bilateral HAs, which were first fitted between 3 and 22 years of age. Hearing loss levels spanned all ranges, including mild (*n* = 3 ears), moderate (*n* = 12 ears), severe (*n* = 3 ears), and profound (*n* = 2 ears). Eight of the HA users had similar hearing levels in both ears (e.g., moderate hearing levels bilaterally), but two had different hearing levels across ears (i.e., mild in one ear and moderate in the other; moderate in one ear and severe in the other). The CI group (*n* = 10, 70% female), whose age ranged from 20 to 48 years, reported the first hearing aid fit between 4 and 32 years of age and first CI activation between 3.5 and 47 years of age. All wore at least one CI (4 bilateral CIs, 5 unilateral CIs, 1 bimodal CI + contralateral HA) and had post-lingual hearing loss in the contralateral ear. The researchers excluded participants with TH in the contralateral ear or those wearing electroacoustic CI devices. The participant who wore a bimodal device configuration completed testing with only their CI.

### 2.3. Data Collection

Because data collection spanned 2018–2021, researchers initially tested participants in person and switched to remote data collection due to pandemic-related research restrictions after March 2020. Fowler [52] completed a validation study comparing performance by five TH participants on in-person versus remote data collection procedures. Results confirmed strong correlations between in-person and remote scores for accuracy on familiar melody recognition (*r* > 0.90) as well as instrument identification, unfamiliar melody accuracy, and pitch discrimination (*r* > 0.70), supporting the sufficiency of collapsing data across in-person and remote collection procedures.

### 2.4. Audiologic Testing

TH participants tested in person underwent a hearing screening to ensure audiometric thresholds of 25 dB HL or better at 500, 1000, 2000, and 4000 Hz in both ears. Two TH participants tested remotely were licensed audiologists who completed hearing screenings in their workplaces and reported TH thresholds to the first author. Participants using HAs or CIs tested in person were screened for aided thresholds of 35 dB HL or better using soundfield stimuli. During the change to remote testing, reliable hearing screening apps could not be determined for individuals with known hearing loss wearing auditory technology. Therefore, this requirement was removed for the remote testing phase, and participants were instead asked how recently they had been to the audiologist to determine likelihood of up-to-date MAPping.

### 2.5. Demographic Characteristics

Participants completed an ad hoc questionnaire querying demographic, audiologic (e.g., diagnosis and severity of hearing loss, history of device use, and current device configuration), and musicianship (e.g., instrument training and experience) history via the online survey platform, Qualtrics. Because the definition of musicianship varies in the literature [53], the authors operationalized it based on the participant-reported ability to read sheet music. That is, participants who currently or previously could read sheet music were considered to have some musical training.

### 2.6. Behavioral Measures

Participants completed three subtests of the Clinical Assessment of Music Perception [34,35]—*Pitch discrimination, familiar melody recognition,* and *instrument identification*—and one subtest of the Profile of Music Perception Skills (PROMS) [54]—*Unfamiliar melody discrimination.*

The *pitch discrimination* subtest uses a two-alternative forced choice paradigm to determine how close in frequency two notes can be before a listener cannot tell them apart. Participants listen to two notes and indicate the note higher in perceived pitch. Scores range from 0.5 (i.e., the distance between a white and black key on a keyboard) to 12.0 semitones (i.e., an octave), with higher values reflecting poorer performance. The chance score for the pitch discrimination task is 1/2 or 50%. This subtest demonstrated strong test–retest reliability with an intraclass coefficient of 0.85 [35].

The *familiar melody recognition* subtest, a closed-set task, asks participants to identify a well-known tune (e.g., *Happy Birthday* or *Twinkle Twinkle Little Star*) presented isochronously to remove the typical rhythmic pattern associated with the melody. Participants were familiarized to each item twice and presented each melody three times in a random order, for a total of 36 tokens. Scores range from 0 to 100%, with larger values indicating better performance. The chance score for the familiar melody recognition subtest is 1/12 or 8.3%. The familiar melody subtest had 0.92 intraclass coefficients during test–retest validation procedures, indicating strong test–retest reliability [35].

The *instrument identification* subtest, a closed-set task, asks participants to identify which of eight instruments was played (i.e., cello, clarinet, flute, guitar, piano, saxophone, trumpet, violin) after familiarization to each instrument. Each instrument plays the same melody previously presented during the familiarization period. The experimental portion plays each melody three times in a random order, for a total of 24 tokens. Scores span from 0 to 100% correct, with larger values indicating better performance. The chance score for the instrument identification subtest is 1/8 or 12.5%. This subtest demonstrated moderate test–retest reliability, with an intraclass coefficient of 0.69 [35].

In the *unfamiliar melody discrimination* subtest [54], participants hear 18 sets of three melodies that include identical stimuli for the first two melodies, but the final melody could present as the same or different. Different melodies vary only in one or more pitches with no variation in rhythm, loudness or instruments. The chance score for the unfamiliar melody discrimination task is 1/2 (i.e., same or different) or 50%. Participants receive one practice stimulus with exaggerated differences among the melodies to confirm understanding of the task. Participants respond with one of five choices: “definitely the same,” “probably the same,” “I don’t know,” “probably different,” or “definitely different.” Scores are assigned based on confidence level, with two points for answers that are “definite” and correct, one point for answers that are “probably” and correct, and no points for “I don’t know” and incorrect answers. The total number of points is summed and divided by two to yield a score that incorporates both accuracy and confidence. This study converted scores to a 100-point scale to allow direct comparison to the *familiar melody recognition* subtest.

### 2.7. Self-Reported Music Abilities and Importance

The *Music-Related Quality of Life* (MURQoL) [10] questionnaire, a 36-item instrument, was developed and validated with 30 adult CI listeners with varying audiologic histories and histories of musicianship. The questionnaire includes two 18-item domains (i.e., *Music Abilities* and *Music Importance*), both of which are subdivided into questions about music perception and engagement (see Appendix A). The *Music Abilities* domain asks listeners to rate their own abilities of perceiving different musical characteristics and listening to music in different situations as a function of how often they feel able to do so on a five-point Likert scale ranging from “never” to “always.” The *Music Importance* domain asks listeners to rate the importance of perceiving different musical characteristics and listening to music in different situations on a Likert scale from 1 to 5, ranging from “not at all important” to “extremely important.” The 18 items in the *Music Abilities* domain mirror the 18 items in the *Music Importance* domain; for example, the *Music Abilities* domain asks participants, “Can you recognize the words in songs?” while the *Music Importance* domain asks participants, “How important is it for you to be able to recognize the words in songs?” This questionnaire was modified only to change UK English spellings to American English spellings. Per questionnaire scoring, the authors transformed scores on the MuRQoL to a 100-point scale, with 0 representing “never”/“not at all important;” 25 representing “rarely”/”not very important;” 50 representing “occasionally”/”somewhat important;” 75 representing “frequently”/”very important;” and 100 representing “always “/”extremely important.” Then, within each domain, averages are calculated to yield a *music abilities* domain score and a *music importance* domain score.

### 2.8. Statistical Analyses

Descriptive statistics were completed for all variables of interest. To address the first two research questions, we performed a multivariate analysis of variance (MANOVA) with hearing status (TH, HA, CI) as the independent variable and behavioral music perception or subjective music perception as the dependent variables, respectively. All statistical analyses used α = 0.05 to determine statistical significance with post hoc testing using Tukey’s tests. Finally, two-tailed Spearman rho (*ρ*) correlations among the behavioral music perception scores and subjective data assessed if relationships exist (a) between the behavioral test scores and the two domains of the MuRQoL, or (b) between the two domains of the MuRQoL (i.e., self-rated music abilities vs. self-rated music importance).

## 3. Results

### 3.1. Descriptive Statistics

Demographic and audiologic variables are presented in Table 1 for the three groups. A total of 40 participants completed all data collection, including 20 TH listeners, 10 listeners with HAs, and 10 listeners with CIs. Two contingency table analyses were run to determine if demographic characteristics differed among the groups. No significant differences emerged for gender representation, *χ*^2^ (12) = 1.25, *p* = 0.53, or musicianship representation, *χ*^2^ (12) = 0.83, *p* = 0.66. Additionally, an analysis of variance omnibus F test revealed no significant main effects of group on chronologic age.

### 3.2. Effect of Auditory Status on Behavioral Measures

A MANOVA was performed to determine if the groups differed in performance on the four behavioral measures (i.e., *pitch discrimination, familiar melody recognition, instrument identification,* and *unfamiliar melody discrimination*). Hearing status significantly influenced overall scores on the behavioral measures (*F* (8, 68) = 7.27, Wilks’ Λ = 0.29, *p* < 0.001), with significant group differences for each of the four behavioral measures.

**Figure 1:**
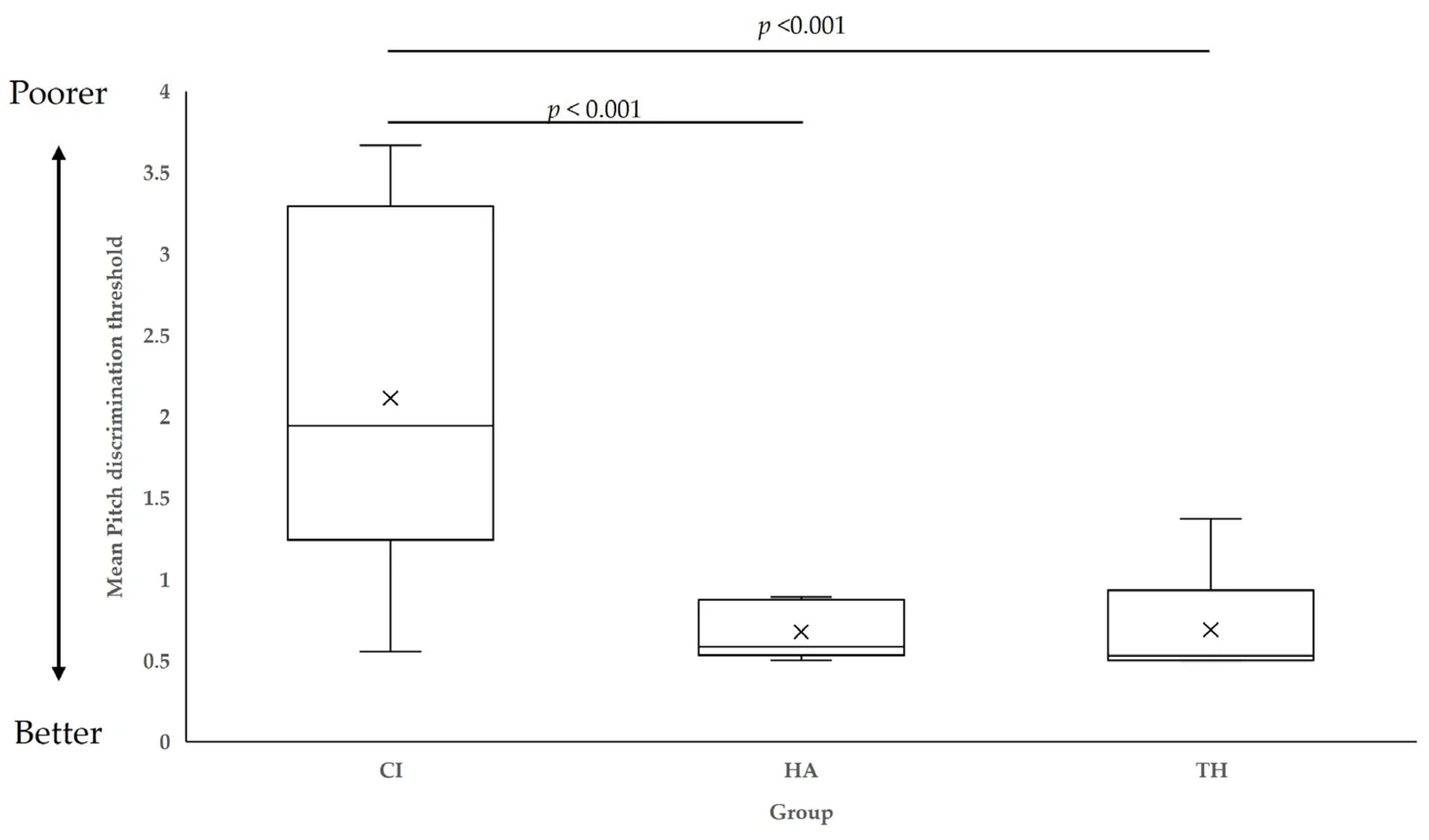
The adult auditory status group on the x-axis and the mean pitch discrimination threshold, measured in semitones, on the y-axis. A lower value on the y-axis reflects better pitch discrimination (i.e., distinction between smaller pitch intervals). The CI group exhibited significantly poorer pitch discrimination than both the TH and HA groups, but the TH and HA groups did not significantly differ from each other.

Group significantly affected performance on the *pitch discrimination* subtest (Figure 1), *F* (2, 37) = 22.76, *p* < 0.001, partial *η*^2^ = 0.552. Post hoc analysis revealed that the CI group (*M* = 2.11, *SD* = 1.10, *Range* = 0.56–3.7) had significantly poorer performance than the other groups, discriminating between pitches when they differed by 1.4 semitones more than both the TH group (*M* = 0.69, *SD* = 0.27, *Range* = 0.50–1.37), with *M* difference = 1.44 (*SD* = 0.26) and *p* < 0.001, and the HA group (*M* = 0.68, *SD* = 0.17, *Range* = 0.50-.89), with *M* difference = 1.42 (*SD* = 0.22) and *p* < 0.001. No significant differences in pitch discrimination emerged among the TH listeners and the HA group, with *M* difference = 0.01 (*SD* = 0.22) and *p* = 0.998.

**Figure 2:**
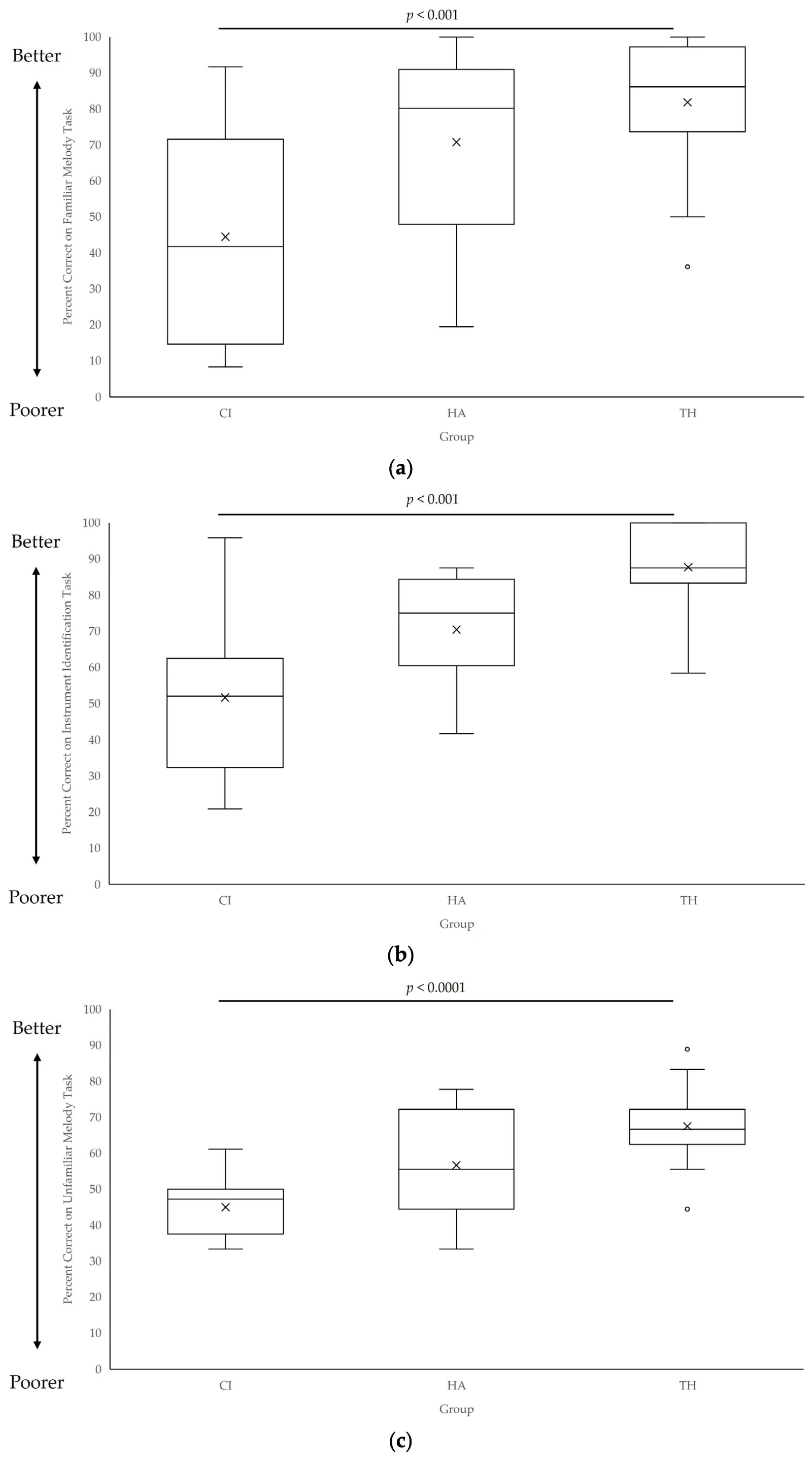
(**a**–**c**) Behavioral measures of music perception (i.e., familiar melody recognition, instrument identification, and unfamiliar melody recognition, respectively) with group (i.e., CI, HA, TH) on the x-axis and the percent of correctness on the y-axis. The CI group exhibited significantly poorer performance on all three subtests compared to the TH group, but no other comparisons reached statistical significance.

On the *familiar melody recognition* subtest (Figure 2a), hearing status significantly influenced performance, *F* (2, 37) = 7.91, *p* = 0.001, partial η^2^ = 0.299. The TH listeners (*M* = 81.81%, *SD* = 18.48, *Range* = 36.11–100%) recognized significantly more isochronous melodies than the CI group (*M* = 44.44%, *SD* = 31.40, *Range* = 8.33–91.67%), with *M* difference = 37.36 (*SD* = 9.40) and *p* < 0.001. No other significant differences emerged between the TH group and the HA group (*M* = 70.75%, *SD* = 26.47, *Range* = 19.44–100.00%), with *M* difference = 11.05 (*SD* = 9.40) and *p* = 0.475, or between the two groups who are DHH, with *M* difference = 26.31 (*SD* = 10.86) and *p* = 0.052.

Hearing status significantly affected outcomes on *instrument identification* (Figure 2b): *F* (2, 37) = 17.43, *p* = 0.001, partial η^2^ = 0.485. TH listeners (*M* = 87.71%, *SD* = 12.38, *Range* = 58.33–100.00%) recognized significantly more instruments than CI users (*M* = 51.67%, *SD* = 21.17, *Range* = 21.17–95.83%), with *M* difference = 36.04 (*SD* = 6.18) and *p* < 0.001, and the HA group (*M* = 70.42%, *SD* = 16.95, *Range* = 41.67–87.50%), with *M* difference = 17.29 (*SD* = 6.18) and *p* = 0.022. In addition, the HA group outperformed the CI group, with *M* difference = 18.75 (*SD* = 7.13) and *p* = 0.032.

Hearing status also significantly affected *unfamiliar melody discrimination*, *F* (2, 37) = 14.10, *p* < 0.001, partial η^2^ = 0.432 (Figure 2c), driven by performance differences by the TH group (*M* = 67.50%, *SD* = 10.08, *Range* = 44.44–88.89%) and the CI group (*M* = 45.00%, *SD* = 8.86, *Range* = 33.33–61.11%), with *M* difference = 22.50% (*SD* = 4.29) and *p* < 0.001. The TH group also outperformed the HA group (*M* = 56.67%, *SD* = 14.53, *Range* = 33.33–77.78%), with *M* difference = 10.83% (*SD* = 4.29) and *p* = 0.04. However, no significant differences emerged between the CI and HA groups, with *M* difference = 11.67% (*SD* = 4.95) and *p* = 0.061.

*Group differences on subjective measures*. Participants significantly differed in their scores on the subjective domains: *F* (4, 72) = 8.12, Wilks’ Λ = 0.48, *p* < 0.001. Tests of between-subjects effects revealed group differences within the domain of *music abilities* (*F* (2, 37) = 17.58, *p* < 0.001, partial η^2^ = 0.487) but not *music importance* (*F* (2, 37) = 0.212, *p* = 0.810, partial η^2^ = 0.011).

**Figure 3:**
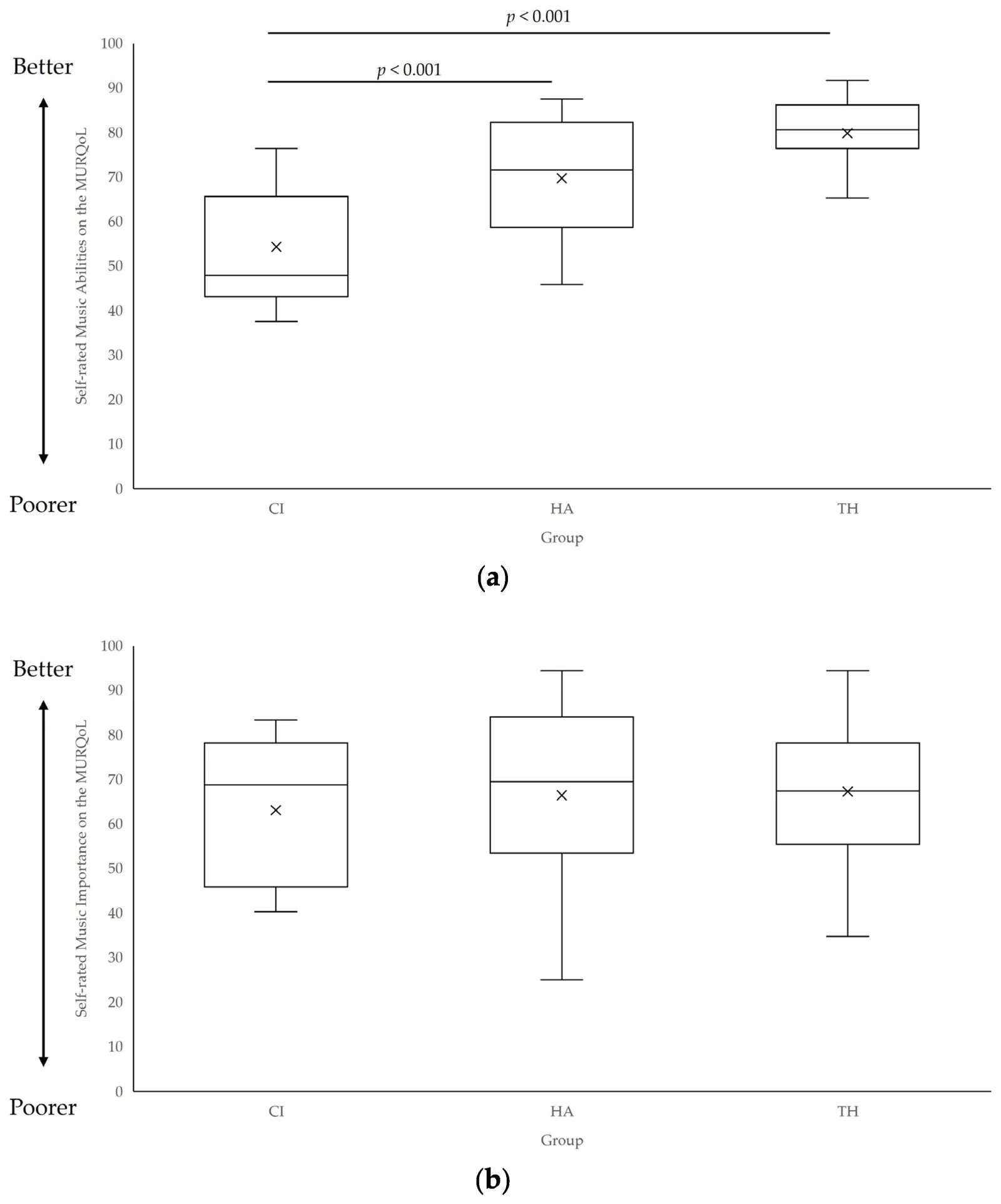
(**a**,**b**) Subjective music subtests (i.e., music abilities and music importance domains in the *Music-related Quality of Life, or MURQoL*, respectively), with group (i.e., CI, HA, and TH) on the x-axis and the self-ratings on the y-axis. The CI group exhibited significantly poorer performance than both the TH and HA groups on the music abilities domain, but the TH and HA groups did not differ significantly from each other. Auditory status did not significantly influence ratings of music importance.

On the *music abilities* domain (Figure 3a), the TH group (*M* = 79.79, *SD* = 7.33, *Range* = 65.28–91.67) rated their own music abilities significantly more positively than the CI group (*M* = 54.31, *SD* = 13.82, *Range* = 37.50–76.39), with *M* difference = 25.49 (*SD* = 4.31) and *p* < 0.001. The HA group (*M* = 69.72, *SD* = 14.30, *Range* = 45.83–87.5) also self-rated their music abilities significantly more positively than the CI group, with *M* difference = 15.42 (*SD* = 4.97) and *p* = 0.01, but not significantly different than the TH group, with *M* difference = 10.07 (*SD* = 4.31) and *p* = 0.063.

On the *music importance* domain (Figure 3b), no evidence of a difference emerged, such that listeners with TH (*M* = 67.24, *SD* = 14.53, *Range* = 34.72–94.44), CIs (*M* = 63.06, *SD* = 16.36, *Range* = 40.28–83.33), and HAs (*M* = 66.39, *SD* = 20.91, *Range* = 25.00–94.44) rated music with similar, moderately high importance (*p* > 0.05).

*Correlations involving behavioral and subjective measures*. Table 2, Table 3 and Table 4 display a correlation matrix for all behavioral and subjective variables for the CI, HA, and TH groups, respectively. Significant correlations emerged between different sets of behavioral measures, but patterns differed across auditory status groups. Better familiar melody recognition significantly co-occurred with better unfamiliar melody recognition for the CI group, *ρ* (n = 10) = −0.759, *p* = 0.011. Better discrimination on the pitch perception task coincided with better familiar melody recognition in the HA group, *ρ* (n = 10) = −0.772, *p* = 0.009. For the TH group, better performance on the pitch perception subtest co-occurred with better familiar melody recognition (*ρ* (n = 20) = −0.651, *p* = 0.002) and better instrument identification (*ρ* (n = 20) = −0.507, *p* = 0.022). In addition, TH listeners with better instrument identification also had better unfamiliar melody recognition, *ρ* (n = 20) = 0.492, *p* = 0.028.

For the CI and TH groups, no significant correlations existed between behavioral music perception and subjective ratings of music abilities and importance. For the HA group, better pitch perception scores coincided with more positive ratings of both music abilities (*ρ* (n = 10) = −0.683, *p* = 0.030) and music importance (*ρ* (n = 10) = −0.705, *p* = 0.023). In addition, the HA users with better familiar melody recognition also rated music importance significantly more positively, *ρ* (n = 10) = 0.648, *p* = 0.043. The relationship between self-rated music abilities and self-rated music importance did not reach statistical significance for the CI group, *ρ* (n = 10) = 0.471, *p* = 0.169, the HA group, *ρ* (n = 10) = 0.517, *p* = 0.126, or the TH group, *ρ* (n = 10) = 0.416, *p* = 0.068.

## 4. Discussion

This study examines the effect of hearing status and hearing technology on behavioral music perception, subjective measures of music abilities and importance, as well as the link between self-reported music abilities and music importance. Results show that adults with CIs have poorer abilities to discriminate pitches, recognize isochronous familiar melodies, identify instruments, and discriminate differences in novel melodies compared to adults with TH or with HAs. Adult HA users and TH listeners do not differ significantly for measures of behavioral music perception. Relative to subjective music abilities, the TH and HA groups self-rate their musical abilities significantly more positively than the CI group, but neither hearing status nor hearing technology significantly influences ratings of the importance of music. Finally, no significant correlations emerge among behavioral music skills, subjective music abilities, or self-rated music importance.

DHH participants in the current study achieved similar levels of pitch discrimination and melody recognition, as reported in the literature. For example, the CI group’s mean performance on pitch discrimination aligns with that of previously studied CI users (i.e., 2.1 vs. 3 semitones) [35,37]. HA users in our study achieved similar levels of familiar melody recognition compared to published findings [24]. Thus, our results converge with the literature for both HA and CI users.

From a group comparison lens, results from the current study confirm previous findings in that listeners with TH achieved significantly better scores than CI users on all behavioral metrics, as expected based on the previous literature for pitch perception [32,33,34,35,36], familiar melody recognition [36], and instrument identification [15,23,34,35,37,39]. We also showed that the TH group performed significantly better than the CI group on the unfamiliar melody recognition task.

However, our findings did not confirm all of our hypotheses. We predicted that the TH group would exhibit significantly better performance on behavioral measures of music perception than the HA group, which would in turn perform significantly better than the CI group. Although results confirmed Simões and colleagues’ [55] finding that TH listeners outperform CI users on timbre and melody tasks, the TH and HA groups showed similarities in scores on all behavioral music perception measures. Equivalent performances by the TH and HA groups contradict findings by Uys and van Dijk [24], who reported that adults with TH scored significantly better on pitch identification, pitch discrimination, melody identification, and timbre perception tasks compared to HA users. In addition, comparisons of the HA and CI groups only partially confirmed our predictions. Compared to Looi et al. [23], who found that adult HA users attained significantly better pitch and melody recognition than adult CI users, our DHH participants only differed significantly on one behavioral task—pitch perception—but they performed similarly on both melody recognition tasks. The discrepancy between Looi et al. and our findings may reflect relaxation of CI candidacy criteria (e.g., current candidates may have more residual hearing and better speech recognition scores), advancements in signal processing (e.g., temporal fine processing), and less traumatic surgical insertion (i.e., preservation of low-frequency thresholds in the implanted ear), all of which have evolved considerably in the interleaving 10 to 15 years between the two studies.

Not surprisingly, the three groups differed on self-rated musical abilities. This is the only metric that confirmed our prediction of graded music perception performance based on hearing status and hearing technology, such that the TH listeners rated their music abilities more positively than the HA users, who rated their music abilities more positively than the CI users. Furthermore, this study suggests that individuals who are DHH recognize their more limited abilities in subjective self-report using the MURQoL, a clinically feasible measure of subjective music experiences that took most participants with HAs or CIs fewer than eight minutes to complete. The MURQoL questionnaire also confirms that despite limited music abilities, participants who are DHH rate music to be just as important as their peers with TH, which aligns with previous studies [10,37,41,42,45,46,47,48].

Neither the CI nor the TH group showed a significant relationship between behavioral and subjective music perception. In contrast, the HA group exhibited significant positive relationships between pitch perception and self-rated music abilities; pitch perception and self-rated music importance; and familiar melody recognition and self-rated music importance. Self-perceived music abilities and music importance did not significantly coincide for any of the groups, which may serve as a testament to the magnitude of the importance of music despite sub-optimal perceptual outcomes.

Because both DHH groups identified music as an important construct, clinicians should support their client’s music engagement by: (a) identifying factors associated with better music perception; (b) modifying programming and MAPping parameters of HAs and CIs, respectively, to maximize access to frequency and intensity; and (c) recommending music training and therapy as part of the rehabilitation program to support satisfactory music perception for adults who are DHH. First, several studies note sociodemographic and audiologic factors associated with better music perception. For example, acoustic input—especially low-frequency acoustic information—in the contralateral ear benefits outcomes for CI users. D’onofrio and Gifford [56] reported better timbre and sound quality with the addition of wideband acoustic information in the contralateral ear. Second, the literature suggests that MAPping strategies improve some aspects of music perception (i.e., processing of multiple simultaneous sounds and melody recognition) with anatomy-based fitting versus default fitting strategies [41,57]. However, the MAPping strategy did not significantly influence self-reported music-specific quality of life—consistent with our findings of the lack of a strong relationship among music perception, satisfaction, and appreciation in adult CI users [41]. Third, music training or rehabilitation may positively influence music abilities. Fuller et al. [58] explored the effect of music-based training paradigms on music perception, finding that programs focused on pitch yielded better melody recognition and programs focused on music therapy resulted in better emotion identification, but neither training program significantly influenced quality of life ratings. Music training can also improve music enjoyment for adult CI users [59,60,61,62]. Gfeller et al. [63] found that experienced CI users favored short-term music training (1–2 weeks) with relatively short sessions (i.e., 30 min). Re-engineered music reflects another way to boost appreciation and engagement. Recent work examined features of music that contribute to enhanced music satisfaction, finding that less complex arrangements with one to three versus multiple instruments [49,64], a shorter reverberation time [65], and fewer harmonic frequencies [66] coincided with higher ratings of enjoyment. Thus, opportunities exist to boost music abilities in adults who are DHH, which, if implemented in a systematic fashion, may influence appraisal of music satisfaction and appreciation in the future. Berg et al. [67] provide clear recommendations for pre-operative counseling, device settings, and programming considerations for adult CI users, emphasizing the necessity to revisit patient performance and satisfaction, particularly for patients for whom music reflects an important part of their life. Additionally, Berg and colleagues suggest clinicians integrate the MURQoL into standard clinical protocols and encourage patients to use rehabilitation training platforms such as Meludia (https://meludia.com) and TeamHearing (https://teamhearing.org/) for music-based exercises.

### 4.1. Strengths and Limitations

This study has several strengths. First, it contributes both behavioral and subjective data on each group, allowing for a fuller picture of experiences with music based on hearing status and hearing technology. While most available studies investigate *either* behavioral [23,24,36] or subjective data [11,27,68] in a wide variety of TH, HA, and CI listeners, rarely are they combined within one study. When both behavioral and subjective data have been collected within one study [37], the undertaking typically excludes the collection of data in comparison groups. The result is a collection of studies that offer valuable insights into how music is perceived in the lives of individuals who are DHH, but which lack similar methodologies that allow for comparison across groups of listeners or types of musical experiences. Thus, inclusion of adults differing in hearing status and hearing technology completing the same behavioral and subjective measures yields a more direct comparison. Additionally, this study provides insight into behavioral testing of music perception and subjective corollaries that could feasibly be done in a clinical setting, thereby addressing issues such as time constraints in adding music perception tasks and questionnaires to clinical appointments without sacrificing timely clinical service.

This study also has limitations. First, a portion of this study was completed in-person, and a portion was completed remotely due to COVID-19-related research restrictions, which may have introduced errors. To ensure as optimal a remote testing environment as possible, the researcher asked participants to be in a quiet room to complete the testing, the researcher paused the testing when disruptions were overheard, and the researcher’s computer was always plugged into an Ethernet port for high internet speed. Although this limitation was not ideal, it did allow the sample to represent a wide geographic distribution (i.e., participants did not need to live in the Dallas-Fort Worth Metroplex to participate in the research), and it ensured participants’ and researchers’ safety during COVID-19 social distancing precautions. Moreover, the first author found a strong correlation between remote and in-person performance by five TH participants, supporting similar performance across the two testing formats [52].

A second limitation centers on the measures used. Available measures of behavioral and subjective music perception may allow for clinical feasibility (i.e., relatively short administration times), but may not fully represent real-world experiences with music. For example, this study did not include rhythm abilities because temporally based tests tend to remain intact for adults who are DHH. Additionally, the MuRQoL was developed with CI users and no studies to date provide norms for HA users.

Third, this study has limitations relative to demographic characteristics and participation classification. This study included a relatively small sample of participants with heterogeneity in key participant characteristics. Thus, their audiologic history (e.g., age at onset, device history, and device settings) and lived experiences may not generalize to the broader community of adults who are DHH. In addition, this study used a crude metric to define musicianship (i.e., reading sheet music), which does not incorporate use of a reliable, valid metric like the Montreal Music History Questionnaire (cite), as used by Zhang et al. [69], or differentiation among musician types (e.g., lyricist vs. instrumental) or playing styles. We should also note that the one bimodal participant completed testing with only their CI, which may not reflect their real-world listening experiences. Also, for the TH group, the authors relied on self-report of typical hearing, which may have introduced errors because 12% of young adults who report typical hearing have measured thresholds >20 dB HL [70].

Fourth, the authors did not collect details on the device characteristics (e.g., HA or CI manufacturer, program settings, or processing strategy) or extended high-frequency audiometric thresholds, which may have influenced music perception. For instance, devices from Advanced Bionics have access to equivalent virtual channels, MED-EL signal processing incorporates temporal fine structure, and Cochlear’s n-of-m strategy emphasizes the most intense sounds or notes in the signal. These differences in signal processing algorithms could affect the individual listener’s perception of music. Furthermore, audiometric testing did not include extended high-frequency ranges or access to low-frequency ranges, both of which may influence spectral resolution and richness, and affect performance on timbre and pitch perception tasks.

Fifth, when participants completed these tests, many expressed frustrations because they suspected they were not scoring very highly. This was especially true for individuals who met the musician description because they could read music. This frustration may have impacted their ability to focus on the tasks that were being presented and may thus have affected final scores. This limitation was mitigated by offering breaks between subtests and discussing with the researcher what was frustrating or useful in the task they just completed.

Future research with larger samples with greater variability in sociodemographic characteristics (e.g., chronologic age or age at onset of hearing loss) and more detailed audiologic and device history may more accurately inform the effects of hearing status and hearing technology on behavioral and subjective ratings of music abilities and importance.

### 4.2. Conclusions

Hearing status influenced outcomes on all behavioral music perception measures for adult listeners. The TH and HA groups performed similarly and outperformed the CI group on two behavioral measures: pitch discrimination and familiar melody recognition. A graded pattern emerged for instrument identification, unfamiliar melody recognition, and self-rated music perception such that TH listeners attained significantly better scores than the HA users, and HA users achieved significantly better scores than the CI users. Ratings of music importance did not differ by group; only those related to behavioral measures in HA users differed. Because adults who are DHH express similar levels of the importance of music to peers with TH, clinicians and researchers should accommodate both behavioral and subjective aspects of music into their interactions with adults who are DHH.

## Figures and Tables

**Table 1:** Participant demographic and audiologic characteristics.

Participant Characteristics	TH ^1^ Listeners (*n* = 20)	HA Users (*n* = 10)	CI Users (*n* = 10)
Mean chronologic age (years)	27.3 (4.6) ^2^	26.0 (5.1)	30.8 (8.4)
Mean age at first device fit (years)	-	10.0 (7.2)	13.4 (10.0)
Mean age at first CI activation (years)	-	-	23.1 (12.3)
Mean duration of device use (years)	-	15.9 (9.1)	7.8 (6.7)

^1^ TH = typical hearing; HA = hearing aid; CI = cochlear implant. ^2^ Values in parentheses denote standard deviations.

**Table 2:** Spearman correlations between behavioral and subjective measures for adult CI users.

	Pitch Perception	Familiar Melody Recognition	Instrument Identification	Unfamiliar Melody Recognition	Subjective Music Abilities	Subjective Music Importance
Pitch perception		−0.614	−0.152	−0.375	−0.361	−0.152
Familiar melody recognition			0.547	**0.759** ^1^	0.552	0.231
Instrument identification				0.617	0.585	0.305
Unfamiliar melody recognition					0.233	0.169
Subjective music abilities						0.471
Subjective music importance						

^1^ Bold values denote statistically significant non-parametric correlations.

**Table 3:** Spearman correlations between behavioral and subjective measures for adult HA users.

	Pitch Perception	Familiar Melody Recognition	Instrument Identification	Unfamiliar Melody Recognition	Subjective Music Abilities	Subjective Music Importance
Pitch perception		**−0.772** ^1^	−0.431	−0.422	**−0.683** ^1^	**−0.705** ^1^
Familiar melody recognition			0.491	0.482	0.444	**0.648** ^1^
Instrument identification				0.602	0.415	0.595
Unfamiliar melody recognition					0.147	0.232
Subjective music abilities						0.517
Subjective music importance						

^1^ Bold values denote statistically significant non-parametric correlations.

**Table 4:** Spearman correlations between behavioral and subjective measures for adult TH listeners.

	Pitch Perception	Familiar Melody Recognition	Instrument Identification	Unfamiliar Melody Recognition	Subjective Music Abilities	Subjective Music Importance
Pitch perception		**−0.651** ^1^	**−0.507** ^1^	−0.314	0.072	−0.142
Familiar melody recognition			0.416	0.429	−0.324	0.057
Instrument identification				**0.492** ^1^	0.312	0.024
Unfamiliar melody recognition					−0.076	−0.033
Subjective music abilities						0.416
Subjective music importance						

^1^ Bold values denote statistically significant non-parametric correlations.

## Data Availability

The datasets presented in this article are not readily available because of limitations of the IRB approval. Requests to access the datasets should be directed to fowlerbrookman@utdallas.edu.

## Abbreviations

The following abbreviations are used in this manuscript:

DHH	Deaf or hard of hearing
HA	Hearing aid
CI	Cochlear implant
TH	Typical hearing
PROMS	Profile of Music Perception Skills
MURQoL	Music-related Quality of Life questionnaire
MANOVA	Multivariate analysis of variance

## Appendix A

**Table A1:** Domains, subscales, and examples from the Music-Related Quality of Life questionnaire.

Domain	Subscale	Definition	*n*	Example Item
Music abilities	Ability-Perception	How well can the subject perceive musical characteristics	11	“Can you recognize the words in songs?”
	Ability-Engagement	How well can the subject engage with music across situations	7	“Do you choose to listen to new music (i.e., music that you have not heard before)?”
Music importance	Importance-Perception	How important is it for the subject to perceive music well	11	“How important is it for you to be able to recognize the words in songs?”
	Importance-Engagement	How important is it for the subject to engage with music across situations	7	“How important is it for you to be able to listen to new music (i.e., music that you have not heard before)?”
